# The Free-movement pattern Y-maze: A cross-species measure of working memory and executive function

**DOI:** 10.3758/s13428-020-01452-x

**Published:** 2020-08-03

**Authors:** Madeleine Cleal, Barbara D. Fontana, Daniel C. Ranson, Sebastian D. McBride, Jerome D. Swinny, Edward S. Redhead, Matthew O. Parker

**Affiliations:** 1 School of Pharmacy and Biomedical Sciences, University of Portsmouth, Old St Michael’s Building, White Swan Road, Portsmouth, PO1 2DT UK; 2 Medicines Research Group, University of East London, London, UK; 3 Aberystwyth University, Penglais, Aberystwyth, Ceredigion UK; 4 School of Psychology, University of Southampton, Southampton, UK

**Keywords:** FMP Y-maze, Zebrafish, *Drosophila*, Working memory, Executive function, Translational research

## Abstract

Numerous neurodegenerative and psychiatric disorders are associated with deficits in executive functions such as working memory and cognitive flexibility. Progress in developing effective treatments for disorders may benefit from targeting these cognitive impairments, the success of which is predicated on the development of animal models with validated behavioural assays. Zebrafish offer a promising model for studying complex brain disorders, but tasks assessing executive function are lacking. The Free-movement pattern (FMP) Y-maze combines aspects of the common Y-maze assay, which exploits the inherent motivation of an organism to explore an unknown environment, with analysis based on a series of sequential two-choice discriminations. We validate the task as a measure of working memory and executive function by comparing task performance parameters in adult zebrafish treated with a range of glutamatergic, cholinergic and dopaminergic drugs known to impair working memory and cognitive flexibility. We demonstrate the cross-species validity of the task by assessing performance parameters in adapted versions of the task for mice and *Drosophila*, and finally a virtual version in humans, and identify remarkable commonalities between vertebrate species’ navigation of the maze. Together, our results demonstrate that the FMP Y-maze is a sensitive assay for assessing working memory and cognitive flexibility across species from invertebrates to humans, providing a simple and widely applicable behavioural assay with exceptional translational relevance.

**Electronic supplementary material:**

The online version of this article (10.3758/s13428-020-01452-x) contains supplementary material, which is available to authorized users.

Neurodegenerative and neuropsychiatric disorders are widespread, causing premature morbidity and increasing social and personal burden (Feigin et al., 2019; Jongsma et al., 2019). These disorders are characterised by diverse cognitive impairments, which can vary significantly within diagnoses, but often have overlapping deficits between disorders (Cope et al., 2016). Impairments in working memory and cognitive or behavioural flexibility are commonly reported in many neurological and neuropsychiatric disorders such as Alzheimer’s disease (Guarino et al., 2019), Parkinson’s disease (Handra et al., 2019; Koerts et al., 2011), schizophrenia (Giraldo-Chica et al., 2018; Orellana & Slachevsky, 2013), depression (Darcet et al., 2016; Hammar & Årdal, 2009; Snyder, 2013), substance abuse (Cunha et al., 2010; Gould, 2010) and autism (Craig et al., 2016; Demetriou et al., 2019). Impairments in working memory and cognitive flexibility have become well-defined behavioural endophenotypes (Harro, 2019; Parker & Brennan, 2012; Wong & Josselyn, 2016) and combined with animal models have become an integral part of translational research (Fontana et al., 2018). However, animal models and behavioural assays have become increasingly diverse, limiting the behavioural fidelity across model species and in clinical findings in human subjects (Day et al., 2008; Young et al., 2009). Therefore, to improve validity of cross-species paradigms there is a need to design assays of executive function that target the same behavioural dimensions or neurobiological measures in a range of species, including humans, to increase validity and translational relevance (Homberg, 2013; Markou et al., 2009).

There is a diverse array of experiments used for assessing animal cognition, with mazes among the most popular (Paul et al., 2009). Existing in numerous behavioural paradigms, the maze can be designed to vary in complexity and target phenotype depending on the task parameters (Sharma et al., 2010). The Y-maze is one of the simplest methods and has been used extensively in learning and memory paradigms for both rodent (Arendash et al., 2001; Conrad et al., 1997; King & Arendash, 2002; Lainiola et al., 2014; Ma et al., 2007) and zebrafish (Aoki et al., 2015; Cognato et al., 2012) models. There are two commonly used methods, the two-choice task in which there is a ‘starting’ arm, a ‘blocked’ arm and the ‘other’ arm. In the first trial, the animal is free to explore, and upon entry into the unblocked arm, is returned to the starting arm. In the second trial, the previously blocked arm is opened. Measurements are recorded for time spent exploring the novel arm (Lalonde, 2002). The alternative method is the continuous Y-maze, in which animals are permitted free exploration throughout the trial, typically lasting 5–8 minutes; the sequence of arm entries is recorded, and working memory capability is determined by the percentage of spontaneous alternation (entry into three different arms in succession) (Hughes, 2004). The Y-maze is proving a useful tool for providing test conditions that do not require rule learning, extensive handling or repeated manipulation (Heredia-López et al., 2016). Other maze tasks, such as the T-maze and radial arm maze, require extensive training, high levels of animal handling and, in reward-based trials, food or water deprivation for prolonged periods (Anderson et al., 2000; Bizon et al., 2007; Deacon, Nicholas, et al., 2006a; Kotagale et al., 2020; Schmitt et al., 2003; Sharma et al., 2010). Each of these factors can result in potential confounders, leading to high levels of between-subject variability (Sharma et al., 2010).

Although valuable, the Y-maze task has several limitations. Some studies have reported difficulties interpreting results, particularly if models tested had hypo- or hyperlocomotion, stereotypic behaviours or anxiety-related novelty avoidance as a consequence of the test condition or treatment, which could significantly interfere with the measurement of spontaneous alternation (Herbert & Hughes, 2009; Hughes, 2004; King & Arendash, 2002; Kumar et al., 2015; Miedel et al., 2017; Stewart et al., 2011). A primary issue, as raised by Stewart et al. (2011) is that a perfect score in the continuous Y-maze, as currently measured, is a reflection of highly stereotyped behaviour. Therefore interpretation of results can be confusing when test models present with repetitive or perseverative behaviours (Cash-Padgett et al., 2016; Miedel et al., 2017).

To address the limitations of current maze methods, we have designed the Free-movement pattern (FMP) Y-maze, a physical maze for animal models and a virtual maze for humans that is analogous to animal versions. The FMP Y-maze is a continuous protocol run using automated tracking software, with built-in data logging of arm entries, aimed at minimising experimenter handling, interference and bias in data interpretation. Our method of data analysis has been developed to allow detail of complex patterns of exploration, using sequences of left and right turns apportioned into 16 overlapping tetragrams (four choices) of left/right combinations ranging from LLLL to RRRR, subsequently shifting the focus away from novelty response to navigational search patterns. Stereotypic responses have been classified as particular search strategies, the presence of which does not overlap with other patterns of normal exploration. Other confounds such as altered locomotor responses are accounted for in the analysis. The use of each sequence pattern is calculated as a proportion of total turns (percentage) and analysed using total turns as a covariate in a general linear mixed model, thus preventing potential inflation of results due to hyperactivity of treatment groups compared to control groups. Prevention of anxiety responses has been diminished by the extension of the run time to 1 hour of free exploration. Not only does this permit a habituation period, but it also removes the need for any pre-trial training, and additionally, the extended trial time allows this method to assess working memory and behavioural flexibility in a single paradigm without having to interfere with any of the task parameters during the trial.

To validate the FMP Y-maze as a measure of working memory and behavioural flexibility, we systemically blocked the glutamatergic, cholinergic and dopaminergic pathways (Blake & Boccia, 2018; Cools & D’Esposito, 2011; K. A. Ellis & Nathan, 2001; Ragozzino, 2002; Ragozzino et al., 2002); dysregulation of these systems has been linked to neurodegenerative and neuropsychiatric disorders (Ballinger et al., 2016; Brisch et al., 2014; Herman & Roberto, 2015; Hindle, 2010; Murueta-Goyena et al., 2017). Additionally, we used time series analysis and autocorrelation to model effects on working memory. Zebrafish treated with antagonists, compared to control groups, demonstrated decreased working memory capacity and changes in search patterns, which were influenced by altered behavioural flexibility. We further validated this task with a range of organisms, including *Drosophila,* mice and humans. Mazes were adapted to suit each organism, but behavioural measures were consistent in all versions. Findings suggested that vertebrate species, zebrafish, rodents and humans, explored in similar patterns, whereas invertebrates adopted an alternative search strategy. Combined, our findings validate the FMP Y-maze as a test of executive function in a range of model organisms, including humans, to create a multifunctional task with high cross-species and translational relevance.

## Experiment 1

Experiment 1 was designed to determine the exploration strategy of zebrafish in the FMP Y-maze. Without prior training or habituation, fish freely explored the novel arena for 1 hour, with continuous recording of arm entries and exits for the duration of the trial. The absence of reinforcement meant that fish did not require periods of pre-trial food deprivation; therefore, fish were taken directly from home tank to test tank, back to home tank, minimising handling and stress in accordance with the 3Rs (Sneddon et al., 2017). Our primary aim was to identify whether the FMP Y-maze could be used as a test of memory. Data from the task were output as a discrete time series (Boyce et al., 2010; Mwaffo et al., 2015), from which we mathematically modelled the randomness of serial observations (Robinson, 2003). Using the two-choice guessing task system introduced by Frith and Done (1983), tetragram analysis was used to identify discernible patterns that departed from a random process (Frith & Done, 1983; Gross et al., 2011). Sequential left and right turns were grouped into overlapping sequences of four turns (tetragrams), giving a total of 16 possible tetragram sequences. The sum of each of 16 overlapping tetragrams of left and/or right turns (e.g. left, left, left, left [L,L,L,L] or right, right, left, left [R,R,L,L]) were analysed to identify strategic search patterns.

### Methods

#### Animals and housing

A total of *n* = 18 zebrafish (*Danio rerio*) of AB wild-type strain (4 months old at time of testing), male and female (~50:50), were bred in-house and raised in the University of Portsmouth Fish Facility. Extensive pilot and published work from our lab has revealed no differences in search strategy between male and female zebrafish ( Fontana, Cleal, & Parker, 2019b). Fish were housed in groups of 8–10 per 2.8 L tank on a re-circulating system (Aquaneering, Inc., San Diego, CA, USA). Sample sizes were calculated based on power analyses (α = 0.05; β = 0.8) from effect sizes observed in pilot studies and previous published work from our group (Cleal & Parker, 2018). Room and tank temperatures were maintained at 25–27 °C on a 14:10-hour light/dark cycle, water was aquarium-treated (dechlorinated) and pH was 8.4 (±0.4). Fish were fed on ZM fry food from 5 days post-fertilisation until adulthood, when they were moved onto a diet of flake food and live brine shrimp (ZM Systems, UK) three times/day (once/day on weekends). On completion of behavioural testing, fish were culled using Aqua-Sed anaesthetic treatment (Aqua-Sed^TM^, Vetark, Winchester, UK) in accordance with manufacturer guidelines.

#### Apparatus

**Fig. 1:**
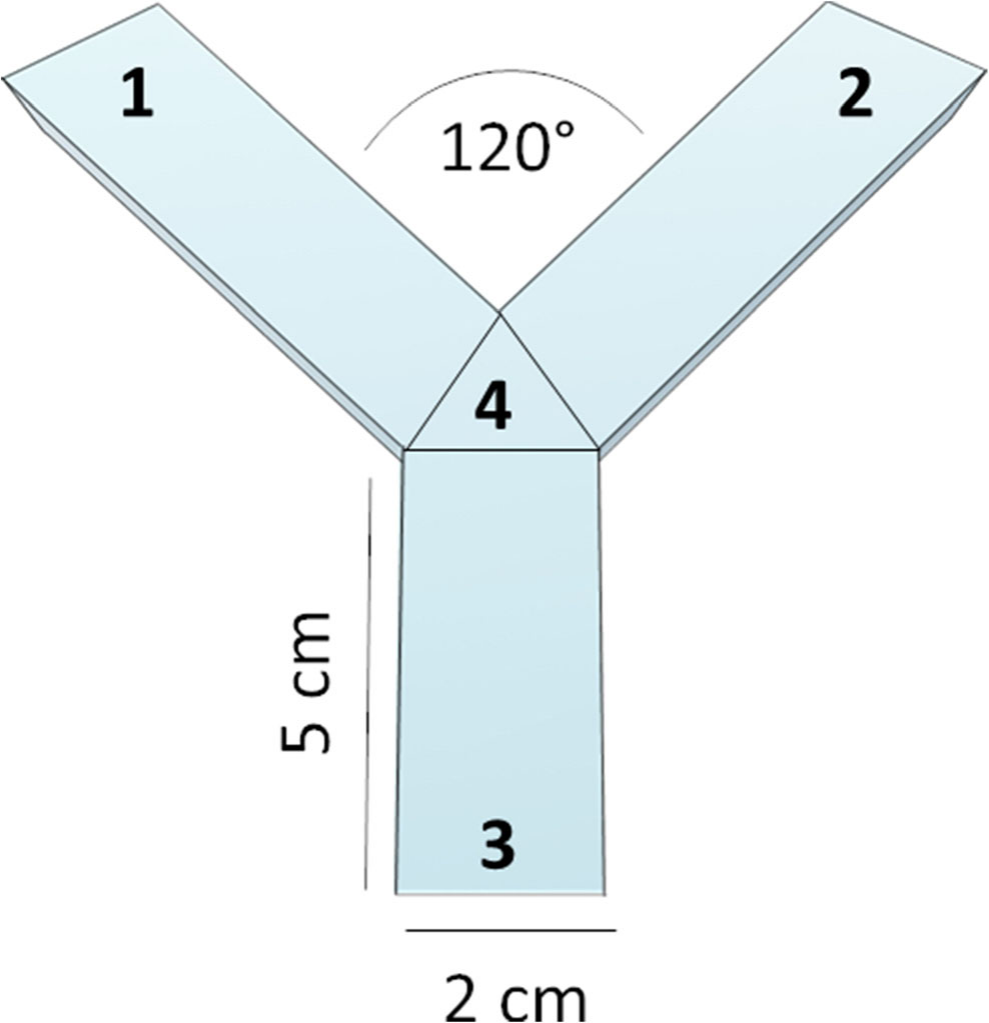
FMP Y-maze diagram depicting maze dimensions and zones used for automated logging of arm entries and exits

**Fig. 2:**
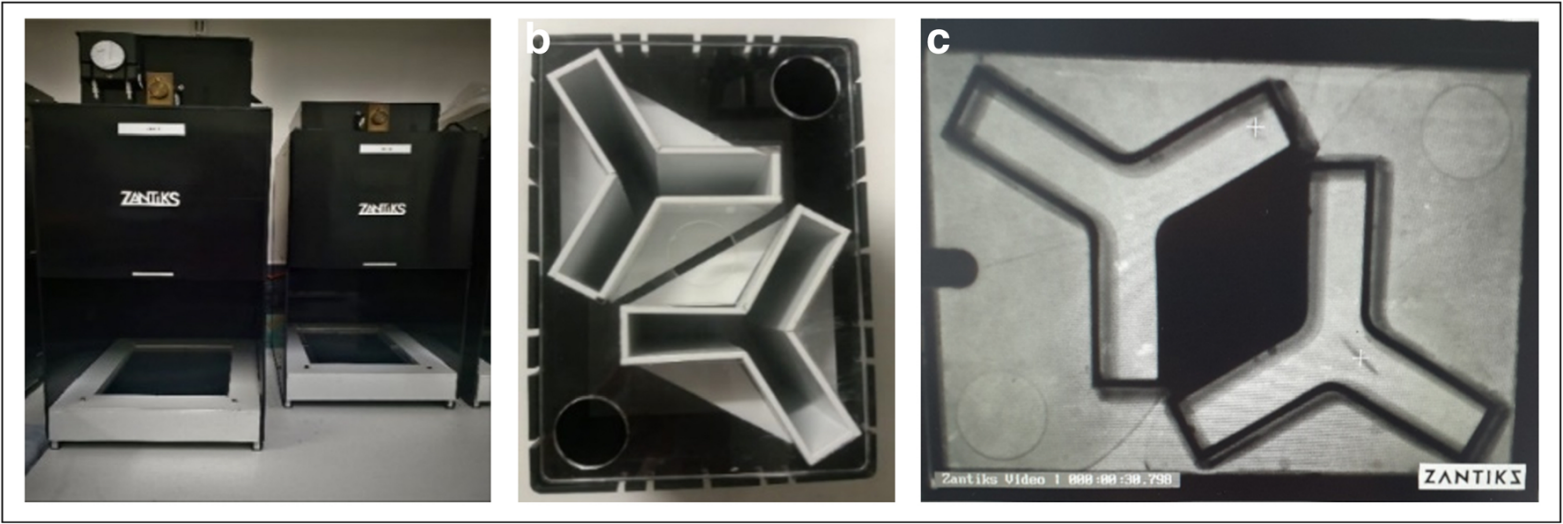
Aquatic FMP Y-maze for zebrafish. (**a**) Zantiks behavioural unit for automated animal tracking. (**b**) Top view of two FMP Y-mazes for zebrafish inserted into a black water-tight tank, L50:W20:H140 mm, filled with 3 L of aquarium water. A mesh lid was used to cover the top of the tank to prevent fish from jumping out during the trial without interfering with the tracking software. (**c**) In trial image of zebrafish in the FMP Y-maze (*n* = 2)

Behavioural testing was carried out in the Zantiks AD system for adult zebrafish (Zantiks Ltd, Cambridge, UK). Zebrafish were tested in white acrylic Y-maze inserts of two identical mazes (provided with the AD Zantiks base package) fitted into a black water-tight tank with a transparent base (https://www.zantiks.com/products/zantiks-ad) (Figs. 1 and 2). Maze dimensions were as follows: L50, W20, H140 (mm). Tanks were filled with 3 L of aquarium water. Each system was fully controlled via a web-enabled device (laptop, phone or tablet). Filming was carried out from above, which allowed live monitoring within the behaviour system (Supplemental video 1). The FMP Y-maze had three equal-sized arms which had no intra-maze cues, although extra-maze (distal) cues were visible from within the maze (e.g. walls and open side of the Zantiks equipment which allowed a small amount of light in). These egocentric cues allow fish to orientate within the maze, but previous studies have demonstrated that these cues do not influence exploratory behaviour (data not shown) (Cleal & Parker, 2018; Fontana, Cleal, & Parker, 2019b; Fontana, Cleal, Clay, et al., 2019a). However, for consistency between tests, light levels were maintained at a consistent level, at a maximum of 2 lux during exploration.

#### Procedure

The protocol was based on that described in our previous papers (Cleal & Parker, 2018; Fontana, Cleal, & Parker, 2019b; Fontana, Cleal, Clay, et al., 2019b). Animal handling and experimenter visibility were both kept to a minimum. Fish were netted directly from home tanks into FMP Y-mazes and inserted into test tanks prefilled with 3 L of aquarium water. Test tanks were placed inside the Zantiks behaviour unit. Water was allowed to settle before starting the protocol to ensure accurate tracking of fish. This step is important, as the initial detection of the animal is crucial to ensure that tracking is accurate throughout the trial. Once the system has successfully detected the animal, a white cross will appear over the animal which will continuously track its movements and log zone entries and exits. Two fish were tested in each behavioural apparatus. Data were initially output as a time series of arm entries and exits, normalised (proportions of total turns) and analysed according to 16 overlapping tetragrams (RLLR, LLRR, RRRL, etc.) (Table 1), of which particular note was taken with regard to search strategies termed alternations (RLRL, LRLR) and repetitions (LLLL, RRRR), having previously seen that these are most notably affected by different treatments. If the fish were adopting a random search strategy, it would be predicted that the distribution of tetragrams over a 1-hour period would be approximately stochastic (i.e. the relative frequency of each tetragram ~6.25%), and the data would generate autocorrelation plots equivalent to white noise (all lagged data points would fall below the 95% confidence interval).

**Table 1:** Tetragram analysis was based on a series of 16 unique, overlapping sequences of left and/or right turns. Below is a list of each tetragram used for analysis, with reference to key strategies and the associated term

Sequence	Term	Step length	Sequence	Term	Step length
LLLL	Repetition	−8	RLRL	Alternation	1
LLLR		−7	RLLR		2
LLRL		−6	RRLL		3
LRLL		−5	LRRR		4
RLLL		−4	RLRR		5
LLRR		−3	RRLR		6
LRRL		−2	RRRL		7
LRLR	Alternation	−1	RRRR	Repetition	8

All experiments conducted for this study were carried out following approval from the University of Portsmouth Animal Welfare and Ethical Review Board, and under license from the UK Home Office (Animals (Scientific Procedures) Act, 1986) [PPL: P9D87106F].

#### Data preparation and analysis

##### Tetragram analysis

In a test paradigm consisting of two equally likely choice variants, left (L) or right (R) turn, we assume choice selection to be completely random. However, we know from human behaviour in guessing tasks (Paulus et al., 1999; Stroe-Kunold et al., 2009), or animals in choice behaviour tasks such as rodents in a T-maze, that there is a preference to alternate L and R turns. Even in paradigms of equal arm reinforcement, choices are never completely random (Deacon, Nick, et al., 2006b; Gerlai, 1998). In a Markov process, a process of completely random events, the probability of choosing L or R depends only on the most recent choice (Grecian et al., 2018). For example, the probability of turning L would be:

$$ \mathrm{P}\left(\mathrm{L}\right) =^{1/2}, $$

regardless of whether the previous turn had been L or R. Despite the overall process being random, it is possible to detect patterns in large data series by dividing sequences into groups of like terms and using information theory to detect any departures from randomness (Meehl, 1993). Let p_*i*_ be the probability of event *i* in a time series, such as the probability of turning L or R. Using general information theory, the first-order ‘uncertainty’ of turning L after previously turning R can be measured using:

$$ \mathrm{L}=\sum {\mathrm{p}}_i{\log}_2\ {\mathrm{p}}_{i,} $$

where base 2 for the logarithm stipulates that from two equally likely events (L or R), one choice (one unit of information) is transmitted to resolve the uncertainty of the occurrence of either choice. Relative uncertainty, L_max_, is the ratio of observed L turns to maximum L turns, for the given number of alternatives; the complement of this is:

$$ 1-\mathrm{L}/{\mathrm{L}}_{\mathrm{max}} $$

Different levels of complexity can be used to determine the probability of turning L based on two previous turns, LR (digram), three previous turns, LRL (trigram), four previous turns, LRLR (tetragram), etc. The larger the number of alternative choices, the greater the computational power required. Previous work has demonstrated that in human guessing tasks, examination of past events exceeding four or five choices becomes irrelevant when calculating the probability of a current event (Hochberg & Attneave, 1961; Meehl, 1993). Therefore, in line with previous two-choice guessing task protocols, we have selected to concentrate on the use of tetragram sequences, limiting the number of alternatives to 2^4^ = 16 possible tetragram sequences. The information measure for a sequence of four turn choices for turning L is:

$$ {\mathrm{L}}_4=\mathrm{L}\left(\mathrm{tetragram}\right)-\mathrm{L}\left(\mathrm{trigram}\right) $$

Tetragram analysis was used to identify patterns over long and short periods of exploration. Tetragram sequences were examined for ‘immediate’ search strategies, i.e. those performed within 10 minutes of exploration, and ‘global’ search strategies that were a consensus of the overall strategy used for the entire hour of exploration. Division of analysis into immediate and global strategies allowed data to be collected on the general exploration strategy and how this strategy was affected by time. This permits examination of multiple characteristics of executive function.

##### Time series analysis

Time series χ_n_= χ_1_, χ_2_…………. χ_k_ were defined as step length, ω(*k*), at discrete time-point, *k*, where *k* was representative of equal-length time points comprised of tetragram sequences. Therefore, each point in the time series was equal to one tetragram, described as one step. Each experiment was made up of *n* time points. The autocorrelation lag coefficients of steps were calculated for each individual using step length, ω(*k*). The autocorrelation function (ACF) was computed in PYTHON using MATLAB (Pal & Prakash, 2017). The lag-1 autocorrelation for the corresponding time lag *k* is:

$$ \mathrm{ACF}(k)=\frac{\sum_{s-1}^{T-k}\left(\omega (s)-\overline{\omega}\right)\left(\omega \left(s+k\right)-\overline{\omega}\right)}{\sum_{s=1}^T{\left(\omega (s)-\overline{\omega}\right)}^2}, $$

where $\overline{\omega}$ is the mean step length for that individuals time series, ω(*k*). As the model demonstrated non-stationary and non-random properties, the usual calculation of confidence interval, $\overline{\omega}\pm 2\sigma /\sqrt{n}$ , where σ is the standard deviation, was not used. Instead, the 95% confidence interval was based on a moving average calculated using the Bartlett test:

$$ T=\frac{\left(n-k\right)\mathit{\ln}{\sigma}_p^2-{\sum}_{i=1}^k\left({n}_i-1\right)\mathit{\ln}{\sigma}_i^2}{1+\left(1/\left(3\left(k-1\right)\right)\right)\left(\left({\sum}_{i=1}^k1/\left({n}_i-1\right)\right)-1/\left(n-k\right)\right)} $$

where ${\sigma}_i^2$ is the variance of the *i*th group, *n* is the total number of steps, *n*_*i*_ is the step length of the *i*th group, *k* is the number of groups and ${\sigma}_p^2$ is the weighted mean of the group variances, defined as:

$$ {\sigma}_p^2={\sum}_{i=1}^k\left({n}_i-1\right){\sigma}_i^2/\left(n-k\right) $$

Tetragram sequences were used to define step length and fix time intervals of the discrete time series. Each sequence was arbitrarily assigned a value ranging from 1 to 8. Left-dominant sequences were arbitrarily denoted as negative, whilst right-dominant sequences were positive (Table 1), from this point on referred to as ‘steps’. Each step was assumed equal time; therefore, each observation in the time series was one tetragram sequence or the equivalent of one step. The analysis for zebrafish was based on 1000 arm entries, sequentially divided into overlapping sequences of four arm entries, resulting in a total of 250 steps, *n* = 250 time points. The limit was chosen arbitrarily for consistency only as total turns varied between individuals. Animals with more than ten steps of missing data were excluded from subsequent time series analysis. Animals with fewer than ten missing steps had zeros replacing missing values to make up the total number of steps required. The cumulative sum of steps was used to determine the relationship between successive observations and identify whether steps were taken randomly and completely independent of one another. This was tested by computing the lag plot and autocorrelation function (ACF) using a custom-designed script in MATLAB.

### Statistics

All turn choices recorded in the FMP Y-maze were converted into tetragrams using customised Excel spreadsheets. Each tetragram sequence was reported as a percentage of total turns completed in the allotted trial time. Based on previous research, alternation (LRLR, RLRL) and repetition (RRRR, LLLL) sequences were analysed independently as dependent variables, as these were the most commonly observed amongst all species. Data were fitted to a general linear mixed effects model (GLMM), with ‘time’ as a within-subjects factor, ‘total turns’ as a covariate to control for general activity levels in statistical models, and ‘ID’ as a random factor. Significant effects were followed by Tukey’s post hoc multiple comparisons test in which each organism was compared to all other organisms. Alpha values of *p* ≤ 0.05 were considered statistically significant. Data are presented as mean ± standard error of the mean (SEM).

### Results and discussion

**Fig. 3:**
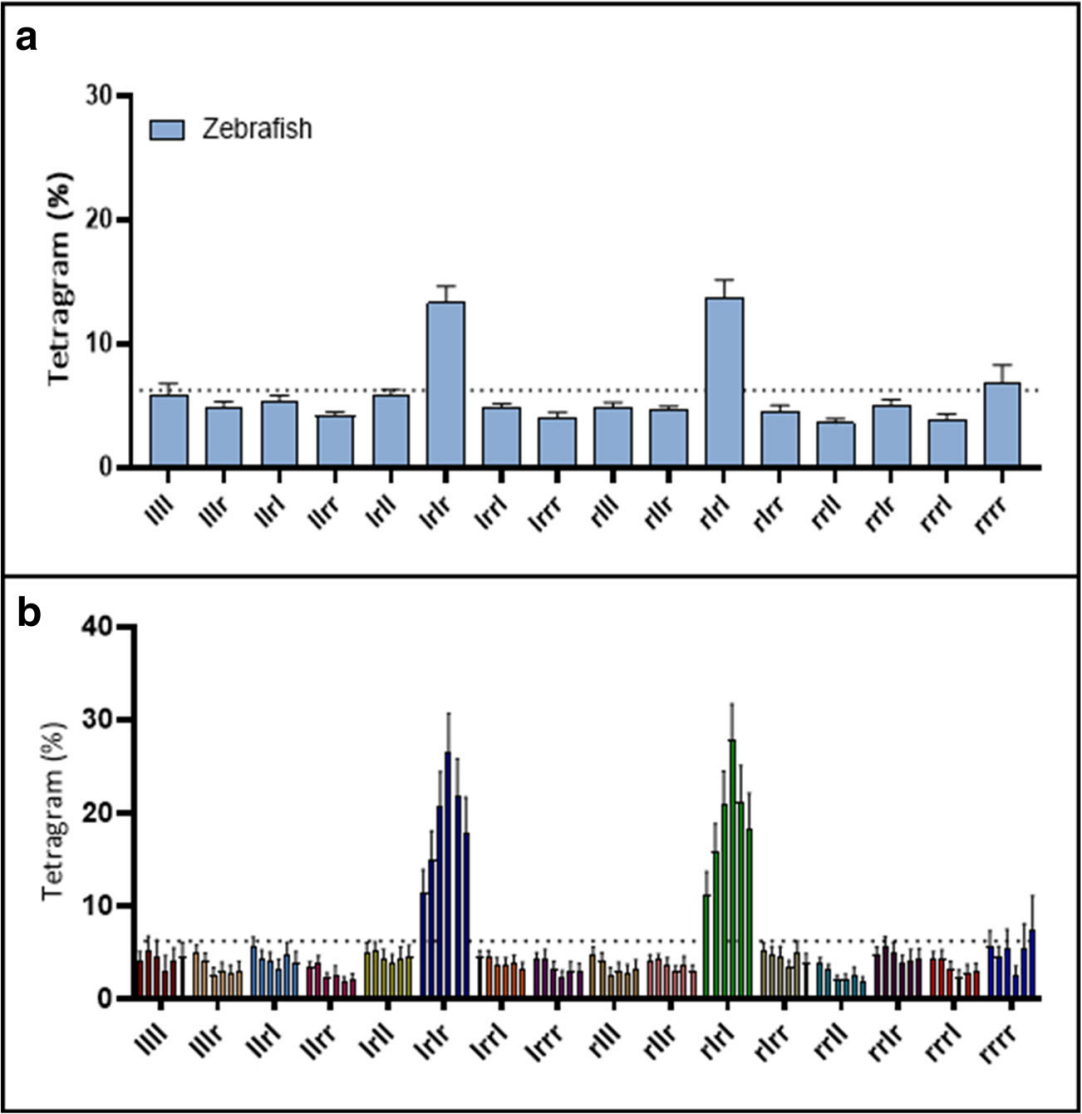
(Top) Frequency distribution of global tetragram strategy over the course of 1 h exploration in the FMP Y-maze (*n* = 18). The dashed line represents random selection at 6.25%. Dominant strategy uses alternations (LRLR, RLRL). (Bottom) Use of each tetragram sequence in 10-minute time bins, demonstrating a clear dominant use of alternations throughout the trial that fluctuate over time. Error bars represent mean ± SEM

Analysis of tetragram sequences used as a global strategy (over the course of the entire trial) revealed that adult zebrafish demonstrated use of a strategy that was significantly dependent on tetragram sequences containing alternating left and right turns (LRLR, RLRL), referred to as alternations (one-way analysis of variance [ANOVA]: *F*_(15, 272)_ = 17.31, *p* < 0.0001; *n* = 21). Although similar to the alternating pattern from the T-maze, in the FMP Y-maze alternations were not used exclusively (which might be consistent with stereotypic behaviour), but instead were distributed regularly throughout the trial (Fig. 3). Alternations were used as a search strategy ~26% of the time, regularly dispersed with other combinations of the remaining 14 tetragrams. The regular occurrence of a specific type of tetragram, the alternation, indicates a complex level of behaviour in which the preceding trigram sequences LRL or RLR are predictors that the following turn choice will be a R or L turn respectively, demonstrating strong intra-sequence dependencies. Thus, despite the overall probability of turning L or R being equally likely, the use of tetragram analysis has revealed the presence of a repeating pattern within the data, resulting in a deviation from complete randomness.

Although tetragram analysis can be used to identify preferential turn choices and dependency of a choice based on the three preceding turns, it cannot be used to determine the persistence of that dependency. Put simply: for a turn choice at position *i*, to what extent are subsequent future turns influenced? Using the lag-1 autocorrelation function (ACF), it is possible to determine the relationship between successive tetragram sequences and identify whether dependency lasts beyond the tetragram set (Bailey & Thompson, 2006). ACFs that rapidly decay, fluctuating around zero, are indicative of a completely random, or memoryless process (Stadnytska & Werner, 2006), i.e. a Markovian process (Reynolds, 2010). However, as we have demonstrated strong intra-sequence dependency of specific tetragrams, we know that turn choice is not random. However, there is no indication of whether a tetragram can influence future tetragram sequences.

**Fig. 4:**
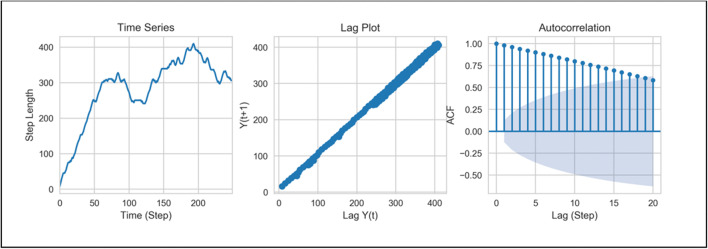
Time series analysis of movement patterns of an individual zebrafish, zf11, (*n* = 1), showing, from left to right, time series plot of the cumulative sum of step lengths for *n* = 250 time points. Lag plot of data at lag-0 (ω(*k*)) and lag-1 (ω(*k*+1)) demonstrating a positive linear correlation. Autocorrelation function plot showing the first 20 lags of 250 lag plots. ACF show slow decay towards zero, with 18 lag points outside of the 95% C.I., depicted by the blue cone. ACF between data points is indicative of dependency between successive turn choices, demonstrating memory of previous events

Our evidence strongly suggests that movement patterns were the result of a global strategy, relying on memory of past turn choices. We therefore hypothesised that subsequent steps (each step representing a tetragram) would demonstrate significant autocorrelation, which would be suggestive of a time series with *memory* of previous events, which exert influence on choice behaviour for a large number of steps. We found that time series plots for individual zebrafish showed either left or right bias, but the ACF of the cumulative sum of steps showed prolonged autocorrelation, which decayed slowly to zero (Fig. 4). These ‘long-range correlations’ between turn choices reflect a long-lasting effect of previous behaviour on subsequent choice behaviour. In sum, our data suggest that the generation of the behavioural sequences of turns by wild type adult zebrafish in the FMP Y-maze are characterised by long-range and significant non-random relationships between steps across a large range of responses.

## Experiment 2

In Experiment 1, we characterised search strategies in the FMP Y-maze and demonstrated that zebrafish rely on working memory to formulate search strategies. To further substantiate the use of memory to navigate the FMP Y-maze we pharmacologically targeted neurotransmitter systems involved in memory processing. The glutamatergic, cholinergic and dopaminergic systems are well documented for their roles in executive functions, particularly working memory (K. A. Ellis & Nathan, 2001; Handra et al., 2019; Myhrer, 2003). Both human and animal studies have demonstrated that pharmacologically blocking these pathways can lead to impairments in working memory tasks (Myhrer, 2003). We hypothesised that blocking NMDA, muscarinic and D_1_ receptors would lead to a reduction in alternations due to impaired working memory. However, as D_2_ receptors are strongly associated with reward and motivation learning and memory processing (El-Ghundi et al., 2007; Kwak et al., 2014), we predicted that pharmacologically blocking D_2_ receptors would not affect search strategy as exploration is conducted in the absence of reward. To this end, we pre-treated zebrafish with low, mid and high concentrations of four antagonists, inhibiting key receptors in the memory process: MK 801, a non-competitive NMDA receptor (NMDA-r) antagonist known to impair working memory by inhibiting long-term potentiation (LTP) (Adler et al., 1998; Lisman et al., 1998; Nam et al., 2004; Nicoll, 2017; Shapiro & Caramanos, 1990); scopolamine, a non-specific muscarinic receptor (M-r) antagonist, similarly to MK-801, reduces LTP in the hippocampus and impairs working memory (Ellis et al., 2005; Granon et al., 1995; Hirotsu et al., 1989), SCH-23390, a D_1_ receptor antagonist and sulpiride, a dopamine D_2_ receptor antagonist (El-Ghundi et al., 2007; Sylvie Granon et al., 2000; Klanker et al., 2013).

### Method

#### Animals

Animals were housed under the same conditions in Experiment 1. A total of *N* = 166 animals were used, with the sample size estimated following power analyses based on range-finding experiments (α = 0.05; β = 0.8). Fish were assigned at random to each treatment group from 10 groups of *n* = 15–20 fish per 6 L tank.

#### Apparatus

The apparatus was identical to Experiment 1.

#### Procedure

##### Pharmacological treatments

To examine the effects of MK801 (Sigma-Aldrich), scopolamine (Sigma-Aldrich), SCH-23390 (Tocris) and sulpiride (Sigma-Aldrich) on performance in the FMP Y-maze, fish were randomly allocated (from > 10 groups of age-matched stocks in our fish facility) to a drug treatment group with ~13 fish assigned per group (*n* = 18 control per drug group; MK801: *n* = 13 0.1 mg/L, *n* = 13 0.75 mg/L, *n* = 13 2.0 mg/L; scopolamine: *n* = 13 0.25 mg/L, *n* = 13 0.5 mg/L, *n* = 13 1.0 mg/L; SCH-23390: *n* = 12 0.5 mg/L, *n* = 12 1.0 mg/L, *n* = 12 1.5 mg/L; sulpiride: *n* = 12 5 mg/L, *n* = 11 10 mg/L, *n* = 11 20 mg/L). Concentrations used were based on previously published works as well as range-finding pilot experiments in our laboratory (Blank et al., 2009; Cognato et al., 2012; Ng et al., 2012; Scerbina et al., 2012; Sison & Gerlai, 2011).

##### Behavioural procedures

Fish were netted from home tanks and placed in 400 mL beakers containing 300 mL of either drug or aquarium water for 1 hour. During pretreatment, fish were visually isolated. This avoided impact of conspecifics or experimenters on treatment response. Following treatment, fish were immediately placed into the FMP Y-maze. Behavioural procedures were conducted in accordance with Experiment 1.

#### Statistical analysis

Tetragram analysis and time series analysis were carried out using the same methods outlined in Experiment 1. In addition, tetragram sequences were fitted to linear mixed effects models, with individual ID as the random effect. Initially, we examined differences in alternations and repetitions. For subsequent analyses, we were interested in putative changes in immediate and global strategies; therefore, ‘time’ was included as the within-subjects factor. To control for variations in general activity levels, ‘total turns’ were included as a covariate in all analyses. The primary endpoint for analysis was the number of choices for each of the 16 tetragrams as a proportion of total turns. Two-way ANOVA was applied separately to the behavioural data obtained from each drug treated group to examine effect of drug concentration on use of alternations and repetitions. ANOVA was followed by Šidák’s post hoc tests (GraphPad Prism 8.4.2). A *p*-value < 0.05 was used as a criterion for significant difference. The data are expressed as mean ± SEM.

### Results and discussion

**Fig. 5:**
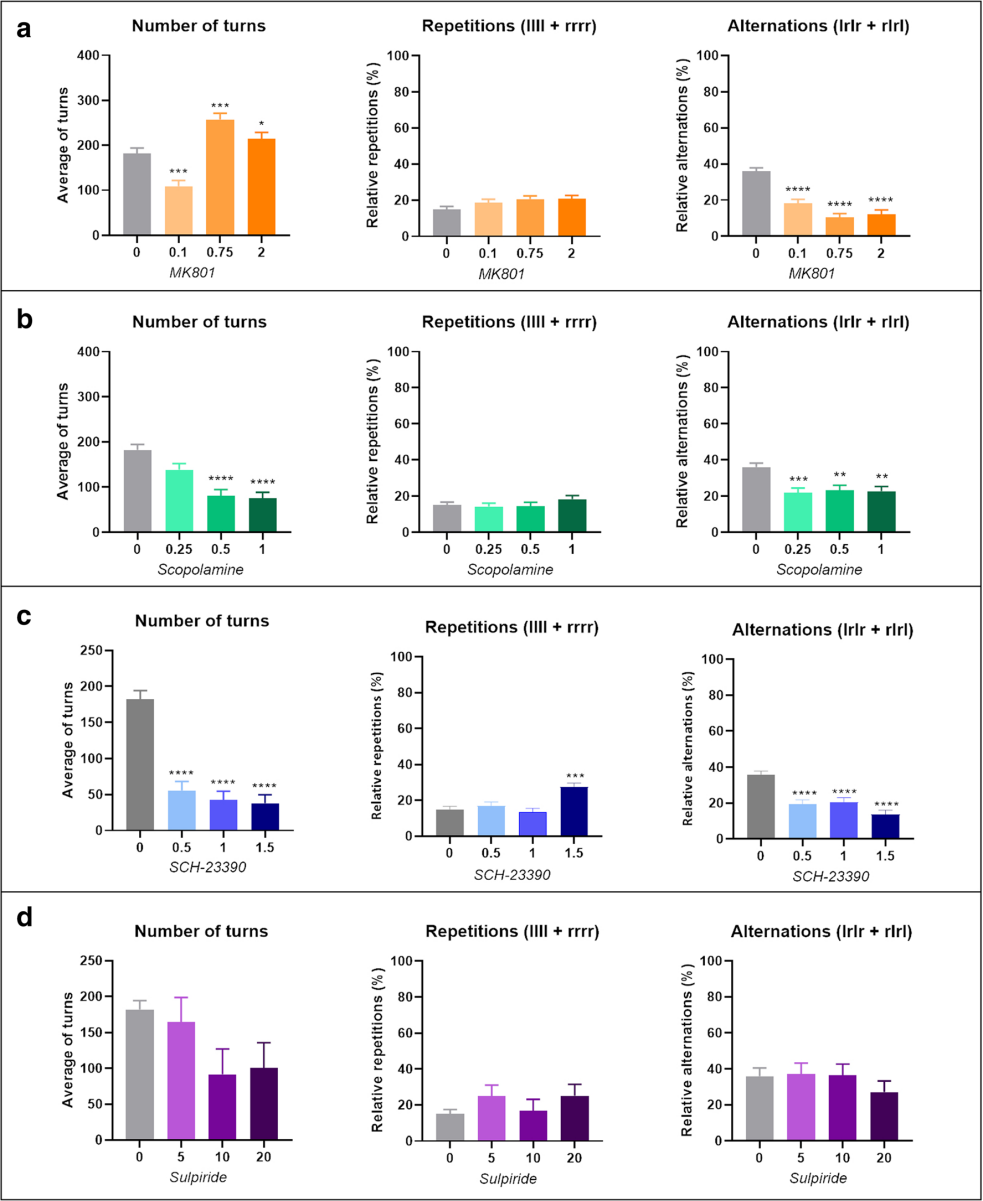
Effects of three concentrations of (**a**) MK 801: control, *n* = 18; 0.1 mg/L, *n* = 13; 0.75 mg/L, *n* = 13; 2.0 mg/L, *n* = 13). (**b**) Scopolamine: control, *n* = 18; 0.25 mg/L, *n* = 13; 0.5 mg/L, *n* = 13; 1.0 mg/L, *n* = 13. (**c**) SCH-23390: control, *n* = 18; 0.5 mg/L, *n* = 12; 1.0 mg/L, *n* = 12; 1.5 mg/L, *n* = 12. (d) Sulpiride: control, *n* = 18; 5 mg/L, *n* = 12; 10 mg/L, *n* = 11; 20 mg/L, *n* = 11) on locomotor activity, in the form of total turns (left), percentage of repetitions used in the global strategy (middle) and the percentage of alternations used as part of the global strategy (right). Data were analysed using a GLMM with total turns as a covariate and ID as a random effect. Bars represent relative frequency of choice, error bars are mean ± SEM. *p < 0.05, **p < 0.01, ***p < 0.001, ****p < 0.0001 compared to control group

**Fig. 6:**
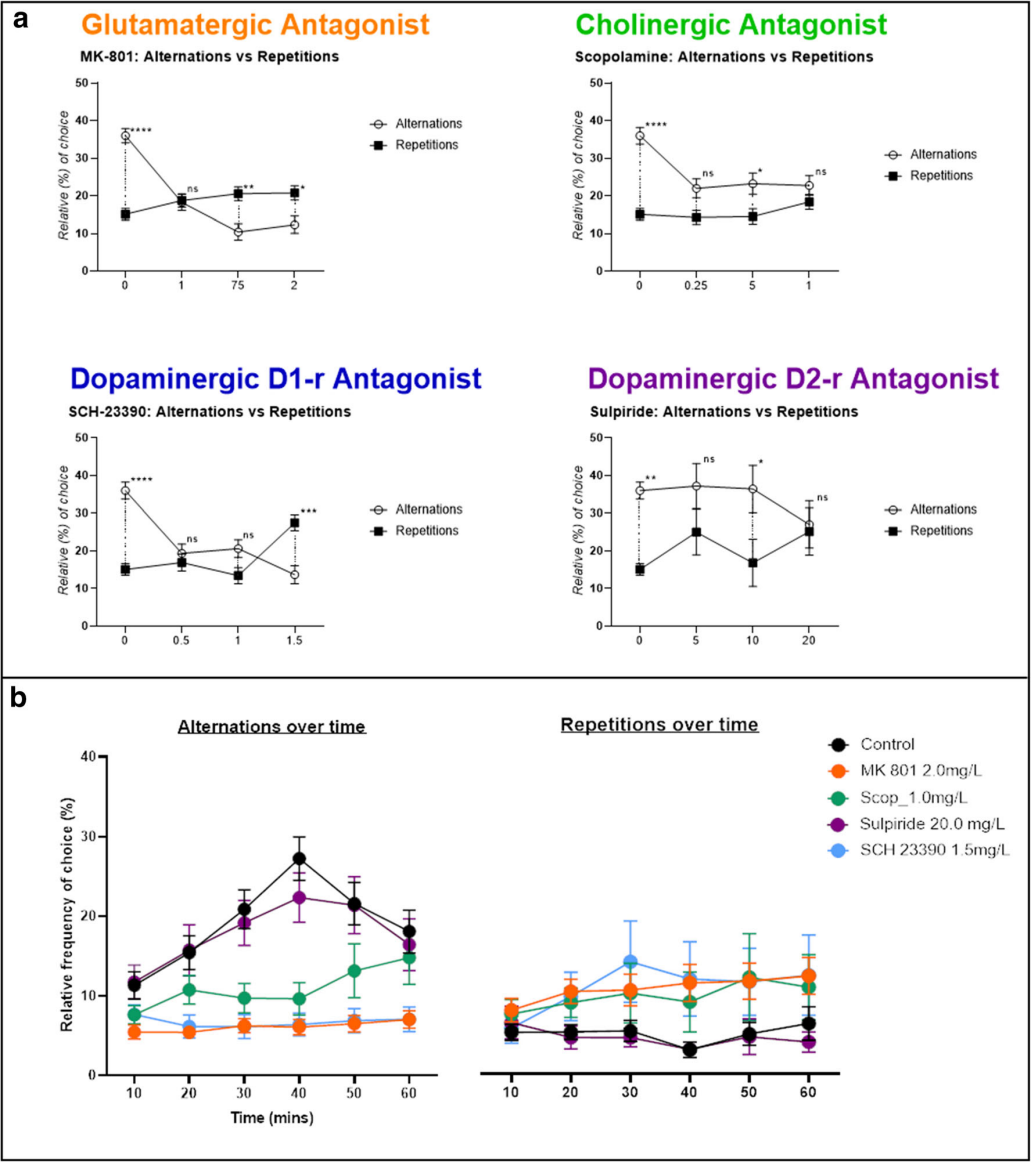
**a** Comparison of total alternations compared to total repetitions for control group (0), low, mid and high concentration of antagonist. Analysis was performed using a two-way ANOVA conducted on the whole data set for each drug treatment separately, followed by Šidák’s post hoc test applied to alternations × repetitions. **b** Change in total alternations (left) and repetitions (right) during 1 hour of exploration divided into 6 equal time bins of 10 minutes per bin. Graphs represent control group versus high concentration of each antagonist treated group. Data were analysed using GLMM. Error bars are mean ± SEM. *p < 0.05, **p < 0.01, ***p < 0.001, ****p < 0.0001, alternations compared to repetitions at each concentration

MK-801 caused a significant decrease in the use of alternations compared to control fish (GLMM, *F*_(3, 318)_ = 34.221, *p* < 0.0001, 0.1 mg/L *n* = 13, 0.75 mg/L *n* = 13 and 2.0 mg/L *n* = 13, control *n* = 18) (Fig. 5a). Chance selection of each tetragram sequence would be ∼6.25%. All concentrations of MK801 reduced alternations to < 6%, effectively blocking alternations as a strategy. In mid and high concentrations of MK 801 (0.75 and 2.0 mg/L) the search strategy was inverted, Šidák’s post hoc test revealed repetitions were used significantly more than alternations (main effect of drug treatment on strategy, *F*_(3, 110)_ = 12.01, *p < 0.001*; Šidák’s post hoc test, 0.1 mg/L alts vs reps, *p =* 0.9994*,* 0.75 mg/L alts vs reps, *p =* 0.0028, 2.0 mg/L alts vs reps, *p =* 0.0182) (Fig. 6a)**.** Scopolamine similarly decreased alternations, but to a lesser extent than MK-801 (GLMM, *F*_(3, 316)_ = 8.025, *p* < 0.0001, 0.25 mg/L, *n* = 13; *p* < 0.001, 0.5, *n* = 13 and 1.0 mg/L, *n* = 13) (Fig. 5b). Post hoc analysis revealed that alternations were only used significantly more than repetitions in fish treated with 0.5 mg/L (main effect of drug treatment on strategy, *F*_(3, 110)_ = 5.01, *p =* 0.0027; Šidák’s post hoc test, 0.25 mg/L alts vs reps, *p =* 0.0728*,* 0.5 mg/L alts vs reps, *p =* 0.0408, 1.0 mg/L alts vs reps, *p =* 0.5443) **(**Fig. 6a**)**. Treatment with SCH-23390, caused two major changes in search strategy. At all concentrations, there was a decrease in the use of alternations, similarly to that caused by MK-801. Additionally, the highest concentration caused an increase in the use of repetitions (LLLL, RRRR) (GLMM test, *F*_(3, 311)_ = 19.692, *p* < 0.0001, 0.5 mg/L, *n* = 12; 1.0 mg/L, *n* = 12; 1.5 mg/L, *n* = 12. GLMM test, *F*_(3, 312)_ = 8.954, *p* < 0.001, 1.5 mg/L, *n* = 12) (Fig. 5c). There was no significant difference between the use of alternations and repetitions at 0.5 and 1.0 mg/L, however treatment with 1.5 mg/L resulted in repetitions being used more than alternations (main effect of drug treatment on strategy, *F*_(3, 110)_ = 6.591, *p =* 0.0004; Šidák’s post hoc test, 0.5 mg/L alts vs reps, *p =* 0.9060*,* 1.0 mg/L alts vs reps, *p =* 0.0993, 1.5 mg/L alts vs reps, *p =* 0.0002) (Fig**.** 6a). No such effect was evident in fish treated with D_2_ antagonist, sulpiride, which resulted in a search strategy resembling control fish (GLMM test, *n* = 33, *p* = 0.622) (Fig**s.** 5d and 6a).

**Fig. 7:**
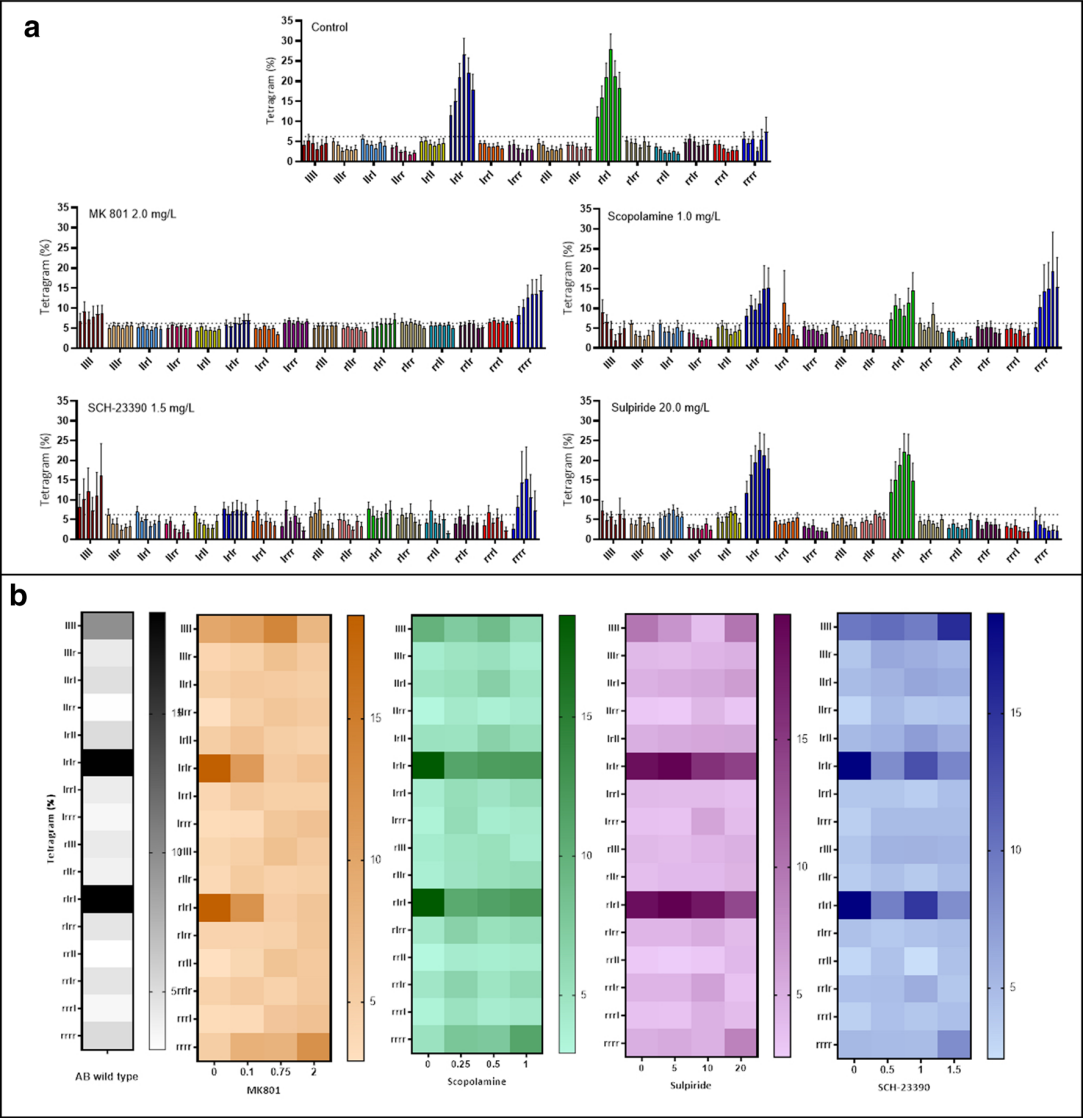
**a** Change in frequency distribution of each of the 16 tetragram sequences as a factor of time; each bar represents a 10-minute time bin. **b** Heat map of changes in global use of each tetragram sequence for each concentration of antagonist compared to control group

The control group showed a clear effect of time on exploration pattern, specifically effecting alternations over successive 10 min search periods (GLMM test, *F*_(5, 186)_ = 5.140, *p* = 0.0002). However, there appeared to be a slight decrease in alternations during the last 20 minutes of exploration. There is no obvious reason for this decrease, and further investigation will be required to examine this change in strategy. MK 801 completely blocked changes in alternation-based search strategy, locking animals in an ‘immediate’ search strategy phase without progression to a global strategy, demonstrating a reduction in behavioural plasticity (GLMM test, *F*_(5, 264.82)_ = 1.499, *p* = 0.191). However, this effect was subject to concentration [*F*_(3, 54.33)_ = 9.70, *p* < 0.001], concentration by time [*F*_(15, 264.81)_ = 2.063, *p* = 0.012] and group interaction [*F*_(1, 54.31)_ = 92.628, *p* < 0.001]. Additionally, MK 801 revealed a significant effect on repetitions over time [*F*_(5, 264.53)_ = 4.36, *p* = 0.001]. Scopolamine reduced alternations in a manner resembling MK 801 treatment. However, inhibiting muscarinic receptors did not have the same effect on impeding behavioural flexibility. Fish treated with scopolamine maintained a significant effect of time on alternations throughout the trial (GLMM test, *F*_(5, 263.79)_ = 4.626, *p* < 0.001), additionally there was an effect of concentration [*F*_(3, 55.41)_ = 2.730, *p* = 0.05], a concentration by time interaction [*F*_(15, 263.62)_=1.897, *p* = 0.024] and group interaction [*F*_(1, 55.53)_ = 141.43, *p* < 0.001], but, unlike MK 801, there was no effect of time on repetitions [*F*_(5, 263.62)_ = 1.936, *p* = 0.089]. Dopamine antagonist SCH-23390 maintained an overall effect of time on strategy [*F*_(5, 259.03)_ = 3.785, *p* = 0.003], however, this effect was disrupted at the highest concentration. Similarly to MK 801, 1.5 mg/L of SCH-23390 blocked the effect of time on alternations [*F*_(5, 60)_ = 0.514, *p* = 0.765]. SCH-23390 also showed an effect of concentration [*F*_(3, 51.98)_ = 5.485, *p* = 0.002], concentration by time [*F*_(15, 259.03)_ = 1.791, *p* = 0.036] and interaction [*F*_(1, 51.98)_ = 105.217, *p* < 0.001]. Finally, the D_2_ receptor antagonist sulpiride resulted in exploration behaviour resembling that of the control group, with a significant effect of time on alternations [*F*_(5, 250)_ = 5.831, *p* < 0.001] and group interaction [*F*_(1, 50)_ = 136.211, *p* < 0.001], but showed no effect of concentration [*F*_(3, 50)_ = 0.594, *p* = 0.622] or concentration by time effect [*F*_(15, 250)_ = 0.686, *p* = 0.798] (Fig. 7).

**Fig. 8:**
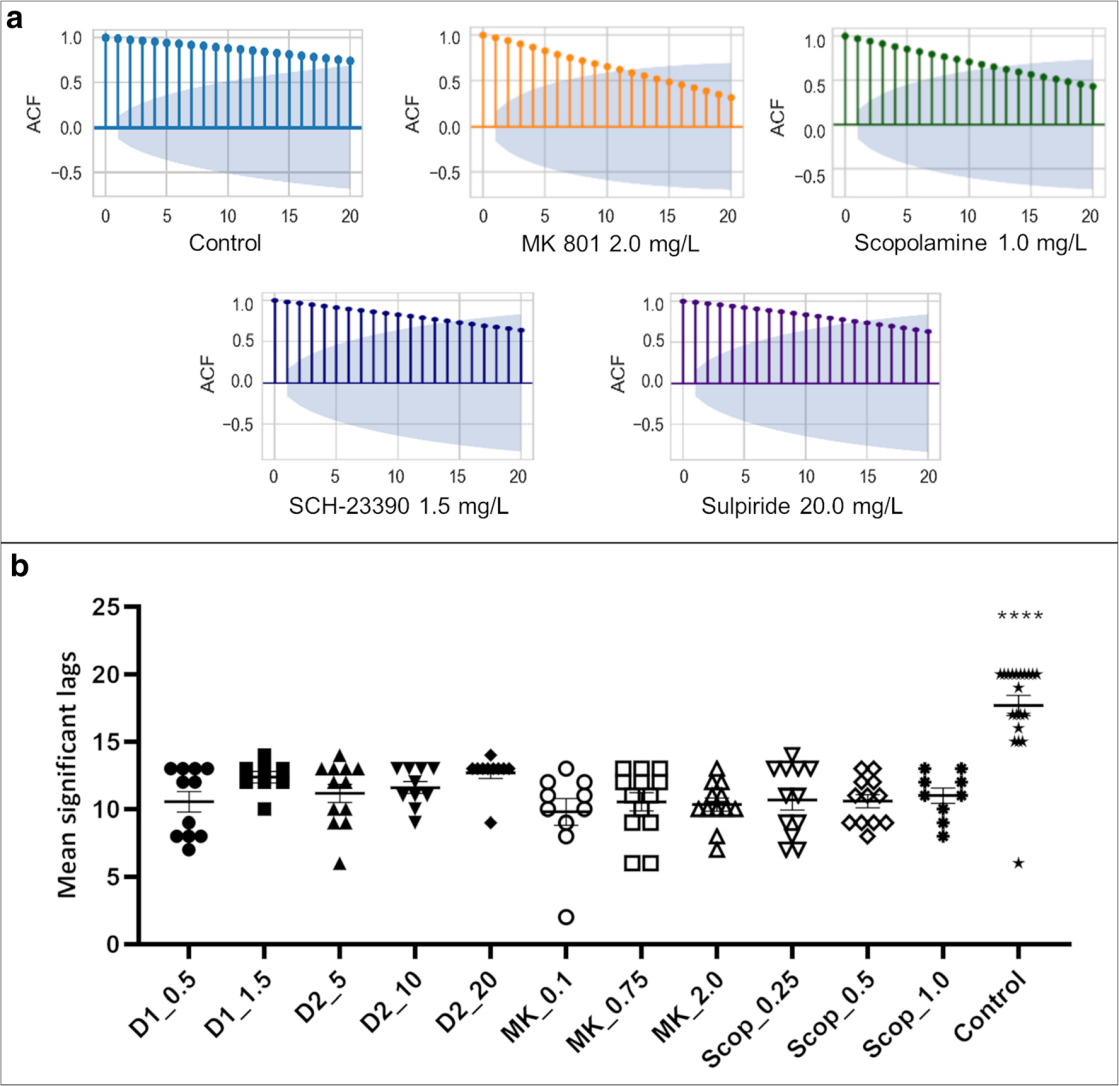
(Top) ACF plot showing the first 20 lags of 250-lag plots. Each plot shows slow decay towards zero, with 18 lag points outside of the 95% C.I., depicted by the blue cone. ACF plots are individual animal responses in the FMP Y-maze and are therefore representative of the control group and drug treatment groups exposed to the highest concentration of antagonist for MK 801, scopolamine, SCH-23390 and sulpiride, respectively. (Bottom) comparison of the mean significant lags of drug-treated groups at low, mid- and high concentrations compared to control group. Bars are mean, error bars are mean ± SEM. ****p < 0.0001, significance is control group compared to treatment groups

ACF plots of each concentration of drug resulted in a decrease in the number of significantly correlated lags compared to control fish (one-way ANOVA; *F*_(11, 127)_ = 13.94, *p* < 0.0001) (Fig**.** 8). Thus, memory impaired zebrafish resulted in shorter-range correlations, limiting the number of steps influenced by choice behaviours showing a reduction in information processing capabilities compared to controls.

## Experiment 3

**Fig. 9:**
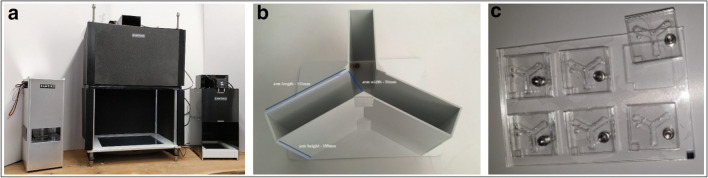
(Left) Zantiks behaviour systems, from left to right, MWP system, LT system and AD system, used for *Drosophila,* mice and zebrafish, respectively. Units are completely automated, with a computer built into the base allowing for image/light projection and a camera positioned above to record live imaging of test animals. This setup reduces experimenter disturbance during testing. (Middle) Mouse Y-maze insert. One mouse per maze. (Right) *Drosophila* Y-maze inserts, six identical mazes with sliding cover to prevent animals from escaping. Six flies can be run per experiment

In Experiments 1 and 2, we demonstrated the suitability of the FMP Y-maze for assessing fish. In Experiment 3 we tested the system with other widely used laboratory species (mice and *Drosophila*). Applying an identical protocol to that used with zebrafish, we characterised the exploration strategies of rodents and flies in the FMP Y-maze using the following apparatus (Fig. 9):

### Methods

#### Animals

##### Mice

A total of *N* = 16 C57BL/6 mice (*Mus musculus)* wild-type (6–8 weeks old at the time of testing), male and female (50:50), were bred in-house and raised in the University of Portsmouth animal facility. Sample sizes were estimated based on power analyses from zebrafish studies (α = 0.05; β = 0.8). Mice were housed in Allentown IVC [individually ventilated cage] racks and kept at 21 °C (±2 °C), 55% humidity (±10%) on a 12:12-hour light/dark cycle. Mice were fed a diet of irradiated SDS RM3 pellets, with food and water available ad libitum. Following use, mice were retained as breeders in the University facility.

##### Drosophila

A total of *N* = 30 Canton S wild-type (#64349) *Drosophila melanogaster* (6–7 days old at the time of testing), male and female (50:50), were obtained from Bloomington *Drosophila* Stock Center, Bloomington, IN, USA**.** Although power analyses for zebrafish and mice showed effect sizes that required *n* = 16, as *Drosophila* had not previously been tested in mazes such as this, we chose to increase the sample size to *N* = 30 to be conservative. Flies were kept at 25 °C, with an average humidity of 60–80%, on a 12:12-hour light/dark cycle**.** Flies were housed on ready-mixed dried food (Phillip Harris, UK). Flies were collected via light CO_2_ anaesthesia and were allowed 48 hours of recovery before behavioural testing was conducted. Following completion of the task, *Drosophila* were culled using absolute ethanol.

#### Apparatus

Mice were tested in a stand-alone white acrylic Y-maze insert with a transparent base (provided with the LT Zantiks base package) (https://zantiks.com/products/zantiks-lt). *Drosophila* were tested in a clear acrylic Y-maze insert of six identical mazes. Each maze had a sliding cover with a hole which could be moved over the maze as an entry point for introducing the fly (extra for the MWP Zantiks unit), fitted into a white opaque holding base for consistent maze alignment (https://zantiks.com/products/zantiks-mwp)**.** Mazes had equal arm length and angle. Maze dimensions were as follows: L152, W50, H155 (mm)-mice, L5, W3, H4 (mm)-*Drosophila*. Mazes were place into their respective Zantiks behaviour units, one maze for mice and six mazes for *Drosophila* (Fig. 9). Systems used worked on the same basis as the AD system used for zebrafish in Experiments 1 and 2. Distal cues and light levels were constant for all experiments.

#### Procedure

Mice were transported from home cage to maze using clear plastic tubes that were kept in their home cages, preventing direct handling prior to the task. *Drosophila* were guided into a pipette tip and tapped gently into the maze through a hole in the lid which could be moved over the maze for entry and, once in the maze, moved away to prevent escape. All animals were recorded for 1 hour. As with Experiments 1 and 2, data were output as a time series normalised as a proportion of total turns and analysed using tetragram sequences. The same statistical analyses were applied from Experiments 1 and 2.

#### Statistical analysis

Two-way mixed-design ANOVA, with one between-subjects factor with three levels (species-zebrafish, mice and flies) and one within-subjects factor with 16 levels (tetragrams), total turns as the covariate, and proportion of choices as the dependent variable, was used to compare global strategies. To examine alternations in more detail, one-way ANOVA determined the difference between tetragram frequencies and as a cross-species comparison of total alternation (LRLR+RLRL) use.

### Results and discussion

**Fig. 10:**
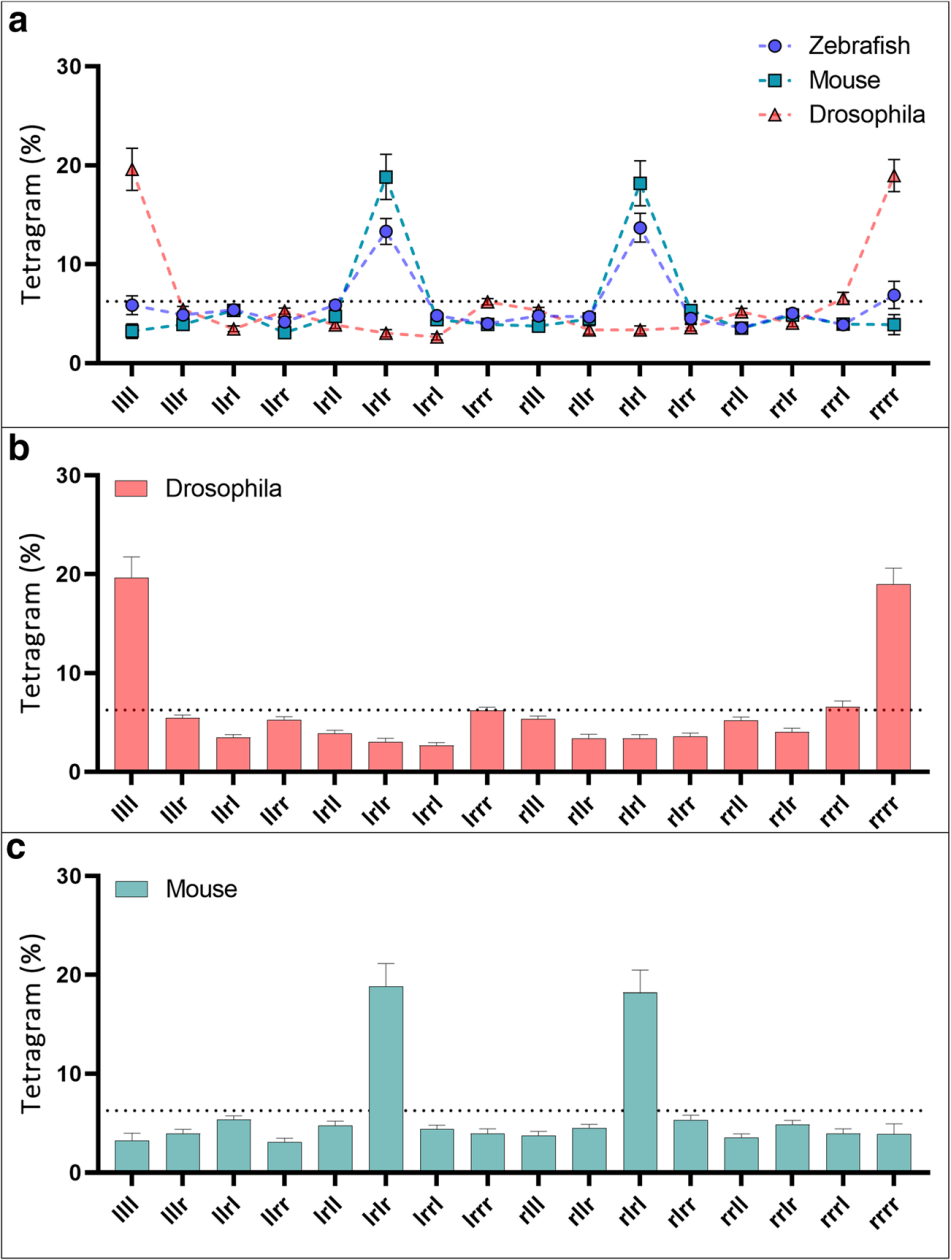
**a** Comparison of zebrafish, mouse and fly global tetragram usage over 1 hour of free exploration. **b** Frequency distribution of global tetragram strategy for 1 hour of exploration in the FMP Y-maze for mice (top, *n* = 15) and **c** *Drosophila* (bottom, *n* = 30). The dashed line represents random selection at 6.25%. Dominant strategy uses alternations (LRLR, RLRL) for mice and zebrafish and repetitions (LLLL, RRRR) for *Drosophila.* Bars represent relative frequency of choice, error bars are mean ± SEM

Mice navigated the FMP Y-maze using an almost identical strategy to zebrafish, showing dominant use of alternations throughout the task (Fig. 10b). There was no significant difference between tetragram frequency distributions for the global strategy (two-way ANOVA, *F*(1, 496) = 1.7^−6^, *p* = 0.999) between mice and fish; however, there was a significant difference in alternations, with mice using alternations ~38% compared to ~26% for zebrafish [*F*(15, 496) = 45.34, *p* < 0.001]. *Drosophila,* however, differed from mice and zebrafish (Fig. 10a), characterised by flies employing an exploration strategy reliant on repetitions as opposed to alternations, which accounted for ~38% of their total search strategy (one-way ANOVA, *F*(7, 472) = 55.12, *p* < 0.001) (Fig. 10c). This alternative navigational pattern could be influenced by *Drosophila’s* natural tendency to explore using wall-following behaviour (Soibam et al., 2012). Like mice and zebrafish, *Drosophila* displayed the dominant ‘repetition’ strategy at evenly distributed times throughout the task, regularly interspersed with different sequences of the other 14 tetragram sequences.

**Fig. 11:**
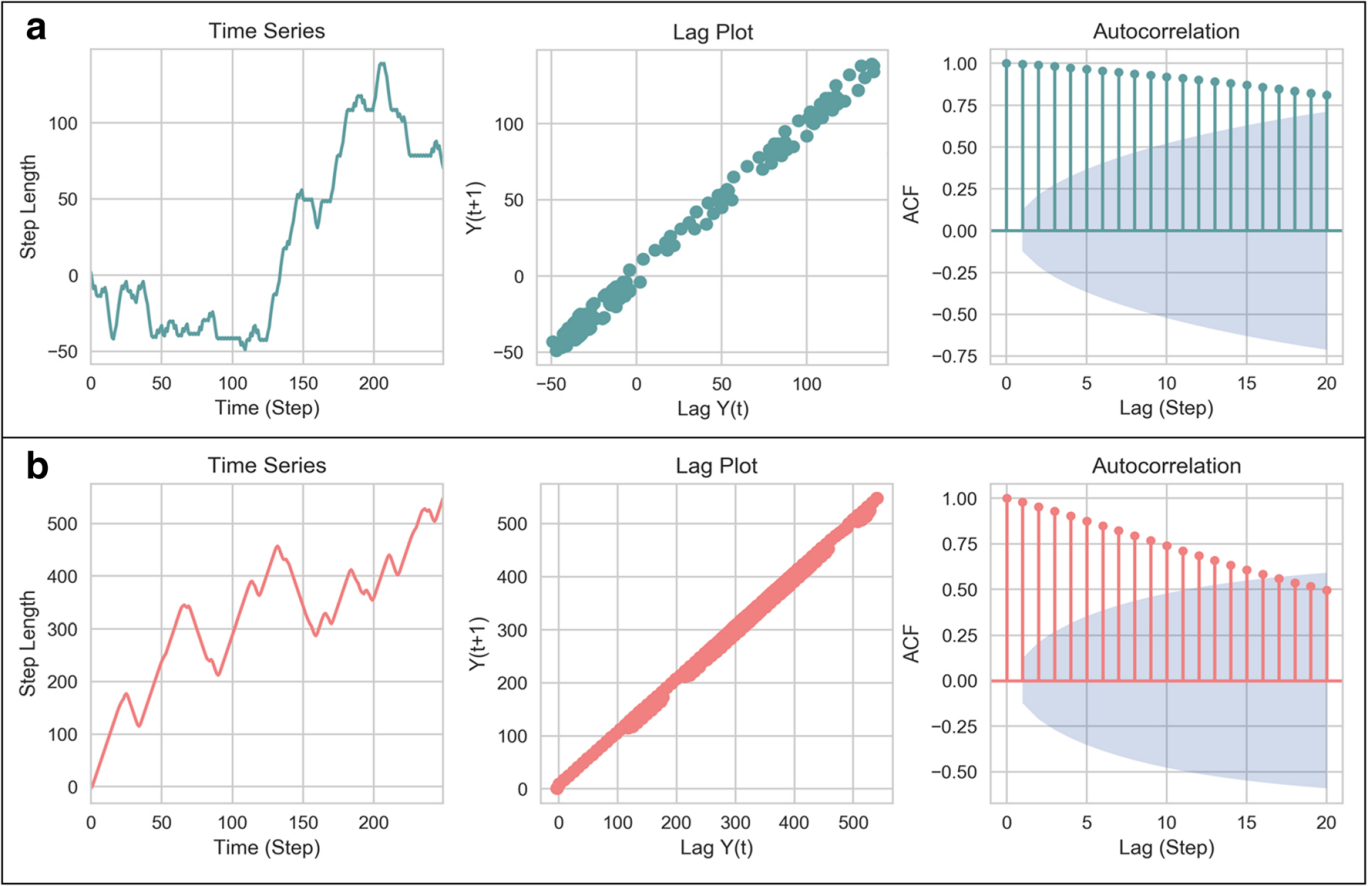
Time series analysis of an individual mouse (top) and fly (bottom) showing time series plot of step length (*n* = 250 steps), lag plot shows a positive correlation for both organisms (middle), ACF plot of the first 20 lag plots both demonstrate over 15 lags of significant autocorrelation

Despite the strategic differences used to explore the maze, all organisms tested showed the use of a single dominant strategy. Regardless of the search pattern, all species showed similar results in the ACF plots, with persistent, slowly decaying autocorrelation, indicative of long-lasting effect of choice on future choice selections (Fig. 11). These data collectively suggest that, like zebrafish, mice and *Drosophila* did not search the test arena randomly, but in a systematic and deterministic way, demonstrating the use of an underlying process of memory to recall previous turn choices and guide subsequent turn patterns. This task provides further evidence of the suitability of the FMP Y-maze as a memory test for a range of model organisms (Supplemental videos 2 and 3)**.**

## Experiment 4

Experiments 1–3 demonstrated the cross-species validity of the FMP Y-maze in laboratory animals: mice, zebrafish and *Drosophila*. In order to test the translational utility of this model, we developed a virtual FMP Y-maze for humans. The maze was based on a honeycomb layout, requiring participants to navigate a series of 'Y’-shaped choice points. In order to make the test clinically relevant and useful for a variety of human testing conditions, we ran the task for 5 minutes, at which point participants were automatically exited from the maze. Previous studies have investigated the relationship between participant response rate and response burden (the perceived effort required by participants to complete an online study, commonly in reference to questionnaires). Increased length of questionnaires has been associated with lower response rates and reduced completion (Presser et al., 2004; Rolstad et al., 2011). In order to increase the translational potential of the virtual FMP Y-maze and suitability to a clinical setting, our aim was to significantly minimise the required participation time in order to reduce boredom, encourage participants to continually traverse the maze for the allotted time and increase the response rate of participants requested to take part in future studies.

### Method

#### Participants

Participants (*n* = 12 male and *n* = 12 female; age range 20-51; mean age = 33.4 ± 8.9 years) were recruited from staff and students at the University of Portsmouth. Sample sizes were estimated from mouse and zebrafish pooled effect sizes (α = 0.05; β = 0.8). Following consent, after reading the information form, participants took part in a short task in which they had to ‘find the way out’ of an online maze. The human experiments were carried out following approval from the University of Portsmouth Science Faculty Ethics Committee (SFEC-2019-062).

#### Apparatus and procedure

##### Human virtual FMP Y-maze

**Fig. 12:**
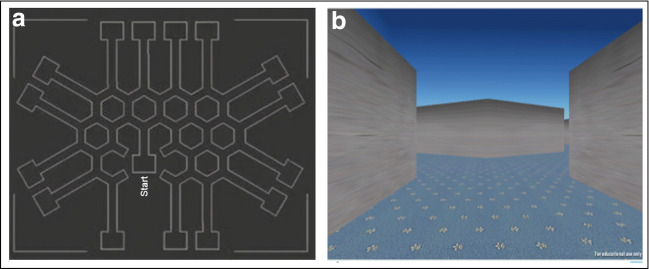
(Left) Schematic of human virtual maze structure showing interconnected Y-shaped mazes, each of equal length and diameter. (Right) Screen shot taken from the human FMP Y-maze from the perspective of the participant, as they explore the maze

A honeycomb maze, representing multiple Y-shaped choice points, formed the human virtual FMP Y-maze (Fig. 12). Participants could initiate the start of the trial when ready and, using the arrow keys on a standard laptop keyboard, navigate their way around the maze (Supplemental video 4). Participants were free to explore the maze for 5 minutes, after which they were automatically logged out. Turn directions were logged as *x*,*y* coordinates, which were converted into left and right turns and subsequently transformed into tetragrams.

### Statistical analysis

To examine the tetragrams, we carried out a one-way within-subjects ANOVA with ‘tetragram’ as the independent variable and proportion as the dependent variable. To example alternations in more detail, two-way ANOVA determined the difference between tetragram frequencies and as a cross-species comparison of total alternation (LRLR+RLRL) use (between-subjects factor – species; within-subjects factor – time).

### Results and discussion

**Fig. 13:**
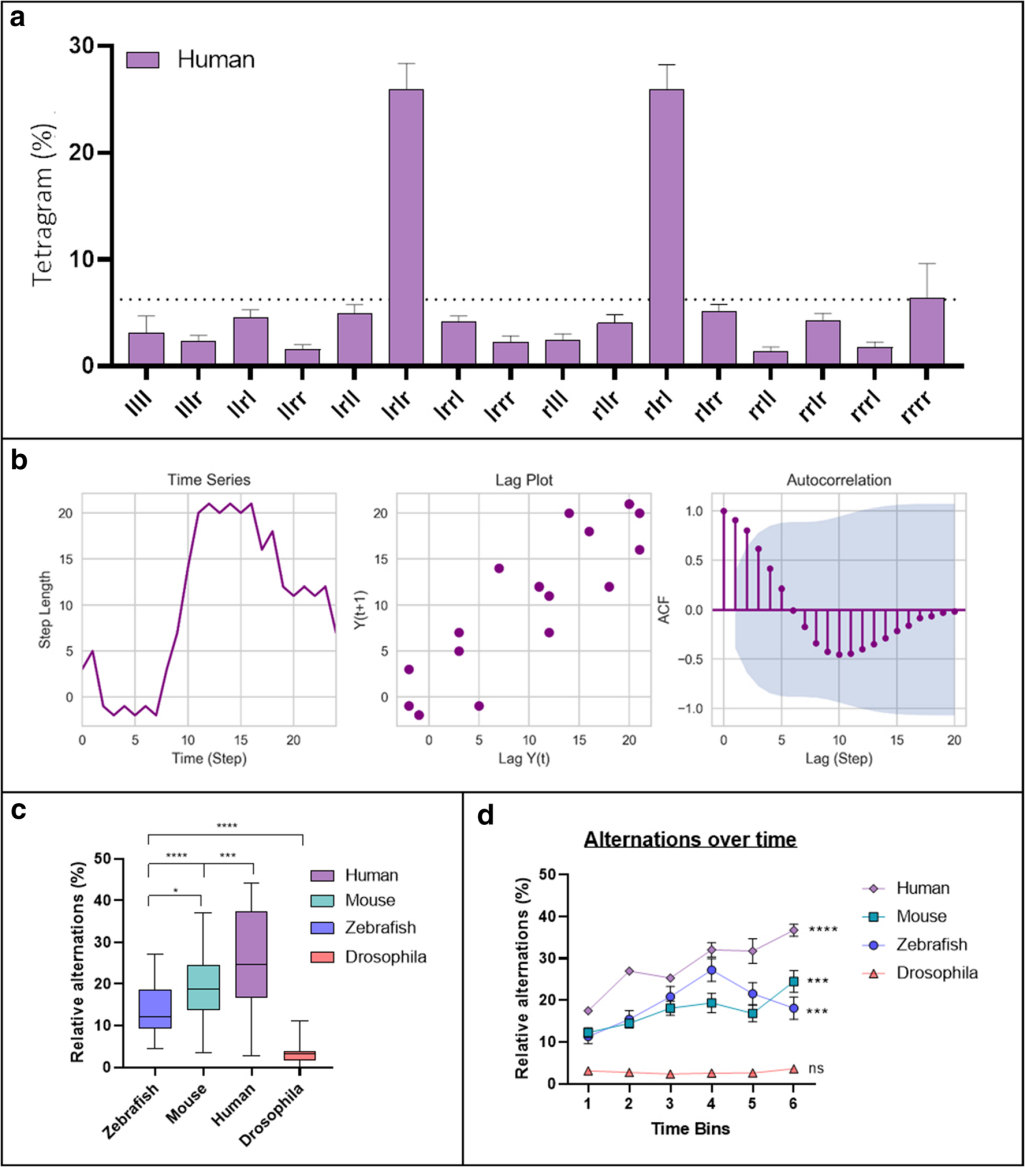
**a** Tetragram frequency distribution of human participants from a 5-minute trial (*n* = 24). **b** Time series analysis of an individual participant showing time series plot (left), lag plot with weak positive correlation (middle) and ACF plot of the first 20 lags, showing significant autocorrelation at lags 1 and 2, which then exponentially decay to zero (right). **c** Relative means of alternations used in the FMP Y-maze of all organisms, demonstrating an increase in percentage use of alternation from zebrafish to mice and peaking with humans. Data were analysed using one-way ANOVA followed by Tukey’s post hoc multiple comparisons test comparing each organism with all other organisms. **d** Alternation used for each time bin (trial time divided into six equal time segments) for humans, mice, fish and flies. Data were analysed by two-way ANOVA, followed by Šidák’s post hoc test comparing time × organism. Error bars are mean ± SEM. *p < 0.05, **p < 0.01, ***p < 0.001, ****p < 0.0001, effect of time on alternations

Tetragram analysis revealed that humans used an almost identical strategy to mice and zebrafish, predominately comprising alternations, which occupied ~50% of the search strategy (one-way ANOVA; *F*_(3, 164)_ = 60.88; *p* < 0.0001) (Fig. 13a). Humans traversing the virtual FMP Y-maze demonstrated significantly greater use of alternations compared to mice, zebrafish and flies (Fig. 13c). Despite limiting the run time to 5 minutes, this prolific strategy was still detectable. On average, participants completed 39 steps (39 tetragrams), with a maximum of 68 and a minimum of 7 steps. The number of steps completed was substantially lower than any of the other animal models and was therefore based on 100 arm entries compared to 1000 arm entries for zebrafish, mice and *Drosophila*. Humans showed a weak correlation in the lag plot and significant autocorrelation lasting only one or two lags before rapidly decaying to fluctuate around zero (Fig. 13b). This indicates that the human FMP Y-maze exploration was characterised by choice selections that were influenced only by the immediate past. Based on the brevity of the trial and the limited number of turns, this would be expected, as the data set was not large enough to determine longer-term patterns. Additionally, there was a significant effect of time on alternations for all the vertebrate species tested (one-way ANOVA: humans, *F*_(5, 6)_ = 19.48, *p* = 0.0012; mice, *F*_(5, 174)_ = 7.635, *p* = 0.0002; zebrafish, *F*_(5, 186)_ = 2.369, *p* = 0.0002), but no effect of time on the invertebrate species (*Drosophila, F*_(5, 342)_ = 1.460; *p* = 0.2994) (Fig. 13d). Our results demonstrate the suitability of the FMP Y-maze as a test of memory, not just for animals, but also for humans, further supporting the theory of a common vertebrate strategy.

## General discussion

We demonstrate that the FMP Y-maze, when combined with tetragram analysis, is an effective tool for assessing executive function, particularly working memory and behavioural plasticity. The ability to detect cognitive impairment in the absence of training, habituation, reward bias or aversive conditions creates a reliable test that can be run singly or as part of a battery of behavioural tasks assessing cognition and memory. The non-invasive nature and low impact on animals provides a task with a strong ‘3Rs’ justification, with particular emphasis on refinement (Tannenbaum & Bennett, 2015). The conserved response strategies across vertebrates demonstrate exceptional, high translational relevance of the task, offering clinical potential.

The FMP Y-maze has implemented the use of an extended protocol which allows 1 hour of free exploration, significantly longer than the 5–8 minutes used for the continuous Y-maze task. The increased runtime provides several advantages: Firstly, as neither the T- nor Y-maze tasks previously included habituation time at the beginning of the trial, it was possible that poor locomotor responses or reduced arm entries were a confound of anxiety in response to a novel environment. The duration of the FMP Y-maze trial permits enough time that persistent behavioural changes can be detected without interference from initial freezing bouts or hypo/hyperactivity. Secondly, exploration patterns more complex than the previously denoted ‘alternation’ strategy, in the continuous Y-maze, can be identified, without ceiling effects. A perfect score in spontaneous alternation tasks is represented by 100% alternations; therefore, it is only possible to detect improvements with this protocol if there is an initial deficit. In comparison, the detection of complex patterns in the FMP Y-maze allows examination of impairments and improvements with, so far, no detection of ceiling effects. Finally, the role of behavioural plasticity can be included as a vital part of the analysis to examine how behaviour evolves over time in response to the environment.

We investigated the role of working memory in flies, fish, mice and humans, in formulating search patterns used to explore the FMP Y-maze. Tetragram analysis revealed two dominant strategies: a vertebrate strategy used by zebrafish, mice and humans that largely consisted of alterations (LRLR, RLRL), and an invertebrate strategy used by *Drosophila* that was reliant on repetitions (LLLL, RRRR). Search behaviour was the result of complex moves that were highly dependent on past turn choices. Time series analysis and autocorrelation revealed that information of previous turn choices was held for long periods, demonstrated by significant autocorrelation for many steps, and used to influence future movement patterns. The length of time this information was held was significantly impacted by pharmacological blockade of glutamatergic, cholinergic and dopaminergic, specifically D_1_, neurotransmitter systems, which showed a decrease in the number of steps with significant autocorrelation. Previous studies in rodents and humans have identified critical roles for each of these systems in maintaining working memory (K. A. Ellis & Nathan, 2001; Handra et al., 2019; Myhrer, 2003). Zebrafish have homologues of each of these neurotransmitter systems (Horzmann & Freeman, 2016), and results from the present study support findings from human and rodent studies of impaired working memory as a result of pharmacologically blocking glutamatergic, cholinergic and dopaminergic receptors (K. A. Ellis & Nathan, 2001; Myhrer, 2003; Shapiro & Caramanos, 1990; Sokolenko et al., 2020; van der Staay et al., 2011), thus highlighting the suitability of zebrafish as a behavioural model for assessing working memory.

Many conditions that commonly report deficits in working memory, such as neurodegenerative or psychiatric disorders, often also present with impaired cognitive or behavioural flexibility (Pittenger, 2013). This represents a change in cognitive state to allow an organism to adapt their behaviour in response to perceived environmental contingencies (Brown & Tait, 2014). In the wild, animals have been found to alter search patterns in response to resources, using one strategy for food-rich areas and another for unpredictable environments with patchy prey distributions (Humphries et al., 2010; Sims et al., 2008). The FMP Y-maze represents an unpredictable environment. Therefore, we would expect animals to alter strategies over time, as has been demonstrated by Namboodiri, et al. (2016), in birds and humans. Cognitively complex organisms have the ability to learn from their environment and subsequently demonstrate modified search strategies when faced with time costs that can reduce the value of a reward or goal (Namboodiri et al., 2016). Here, we show that healthy fish, mice and humans all demonstrate some degree of behavioural flexibility whilst traversing the maze, by increasing the use of alternations over time. However, flies used a strategy that was static throughout the trial and did not differ significantly from the first 10 minutes to the last 10 minutes of exploration. This method has demonstrated sensitivity to detect adaptive behaviours in response to time and the environment in a range of cognitively complex organisms.

Further testing with pharmacological agents demonstrated the ability of this task to detect drug-induced changes in adaptive behaviours. MK 801 has been used in previous studies to model schizophrenia-like behaviours, including deficits in working memory and cognitive flexibility (Lobellova et al., 2013; Murueta-Goyena et al., 2017; Svoboda et al., 2015). Here we demonstrate that the FMP Y-maze protocol could detect impaired behavioural flexibility induced by systemic blockade of NMDA receptors by acute MK 801 exposure. This task could also detect changes in behavioural adaptability after acute exposure to muscarinic and dopaminergic D_1_ receptors, but no effect of systemic D_2_ receptor blockade, in line with findings from previous rodent studies (Chen et al., 2004; Ragozzino et al., 2002; Winter et al., 2009). These results further support the use of the FMP Y-maze to detect changes in cognitive flexibility and the use of zebrafish to model cognitive impairment.

Deficits in executive functions such as working memory and cognitive or behavioural flexibility are commonly reported in patients diagnosed with neurodegenerative diseases such as Alzheimer’s (Guarino et al., 2019) and Parkinson’s disease (Handra et al., 2019; Koerts et al., 2011), or as a feature in a variety of psychiatric disorders such as major depressive disorder (Darcet et al., 2016; Hammar & Årdal, 2009; Snyder, 2013), substance abuse (Cunha et al., 2010; Gould, 2010) and schizophrenia (Giraldo-Chica et al., 2018; Orellana & Slachevsky, 2013). As working memory and cognitive flexibility can be markers for many complex brain disorders, the FMP Y-maze could be used as a clinical behavioural task for assessing executive function and memory processing as part of a battery of diagnostic tools. The ease and brevity of the human FMP Y-maze task lends itself to testing all age groups, including adolescents who may have increased susceptibility to developing schizophrenia (Bossong & Niesink, 2010; Hollis, 1995). Additionally, the neurotransmitter groups tested here have been implicated in a number of neurodegenerative and neuropsychiatric disorders and their treatments (Aarsland et al., 2017; Brisch et al., 2014; Francis, 2005; Li et al., 2019; Murueta-Goyena et al., 2017).

Despite the advantages of testing executive function in the FMP Y-maze, there are limitations to the protocol, primarily associated with run time. Animal versions of the FMP Y-maze are run over 1 hour. Although this provides some benefits, as outlined above, the time taken to run a full experiment is largely dependent on the resources available to the facility. We operated this task with one MWP unit, one LT unit and four AD units. Thus, we were able to run eight zebrafish, six *Drosophila* and one mouse per hour. In total, it took three days of back-to-back trials to test 166 zebrafish, 5 hours to test 30 *Drosophila* and three days to run 16 mice. Therefore, the level of throughput is dependent on the organism being tested and the number of behavioural units available for simultaneous trials. Additionally, this run time could not be applied to the human maze, as the extensive trial time would be expected to have a negative impact on participant recruitment. Therefore, the trial was reduced to 5 minutes of exploration. However, the time for the online trial may need to be amended depending on the target group. Preliminary studies showed that younger participants completed sufficient turns in the allotted time, but that older participants completed very few turns, and for some this resulted in exclusion due to insufficient data collection. Therefore, it was suggested that for studies targeting older groups, or treatment groups with cognitive impairments, that run time be increased.

Here, we present a new behavioural task for testing deficits in executive function and working memory. We demonstrate the reliability and sensitivity of the FMP Y-maze to alterations in cognition and memory processing in a range of model organisms. Additionally, an online virtual maze has been created as a translational cognitive paradigm for testing humans. This task has the potential to be used either as a diagnostic tool or as a method for improving drug discovery using animal models of complex brain disorders that report memory and cognitive decline as hallmarks of disease. The FMP Y-maze lays the foundation for future translational research in a range of neurological disorders and could open new avenues of research into cognition and memory, allowing cross-species comparisons with exceptional translational relevance.

## Electronic supplementary material

ESM 1 (MP4 4927 kb)

ESM 2 (MP4 5315 kb)

ESM 3 (MP4 4288 kb)

ESM 4 (MP4 3805 kb)
